# Initiation of anti-retroviral/Trimethoprim-Sulfamethoxazole therapy in a longitudinal cohort of HIV-1 positive individuals in Western Kenya rapidly decreases asymptomatic malarial parasitemia

**DOI:** 10.3389/fcimb.2022.1025944

**Published:** 2022-11-24

**Authors:** Carolyne M. Kifude, Ashleigh Roberds, Janet Oyieko, Stephen Ocholla, Solomon Otieno, John N. Waitumbi, Jack Hutter, Hunter Smith, Nathanial K. Copeland, Shirley Luckhart, V. Ann Stewart

**Affiliations:** ^1^ Kombewa Clinical Research Center, Kenya Medical Research Institute-United States Army Medical Research Directorate-Africa, Kisumu, Kenya; ^2^ Department of Preventive Medicine and Biostatistics, Uniformed Services University of Health Sciences, Bethesda, MD, United States; ^3^ Department of Entomology, Plant Pathology and Nematology, University of Idaho, Moscow, ID, United States; ^4^ Department of Biological Sciences, University of Idaho, Moscow, ID, United States

**Keywords:** HIV-1, antiretroviral, asymptomatic, malaria, parasitemia

## Abstract

Interactions between malaria and HIV-1 have important public health implications. Our previous cross-sectional studies showed significant associations between HIV-1 positivity and malarial parasitemia with an increased risk of gametocytemia. In this follow-up longitudinal study, we evaluated these associations to determine the magnitude of asymptomatic parasitemia over time, and to examine the effects of initiating Antiretroviral Therapy (ART) together with the broad-spectrum antibiotic Trimethoprim Sulfamethoxazole (TS) on asymptomatic parasitemia. 300 adult volunteers in a malaria holoendemic region in Western Kenya were enrolled and followed for six months. The study groups were composed of 102 HIV-1 negatives, 106 newly diagnosed HIV-1 positives and 92 HIV-1 positives who were already stable on ART/TS. Blood samples were collected monthly and asymptomatic malarial parasitemia determined using sensitive *18S* qPCR. Results showed significantly higher malaria prevalence in the HIV-1 negative group (61.4%) (*p*=0.0001) compared to HIV-1 positives newly diagnosed (36.5%) and those stable on treatment (31.45%). Further, treatment with ART/TS had an impact on incidence of asymptomatic parasitemia. In volunteers who were malaria PCR-negative at enrollment, the median time to detectable asymptomatic infection was shorter for HIV-1 negatives (149 days) compared to the HIV-1 positives on treatment (171 days) (*p*=0.00136). Initiation of HIV treatment among the newly diagnosed led to a reduction in malarial parasitemia (expressed as *18S* copy numbers/μl) by over 85.8% within one week of treatment and a further reduction by 96% after 2 weeks. We observed that while the impact of ART/TS on parasitemia was long term, treatment with antimalarial Artemether/Lumefantrine (AL) among the malaria RDT positives had a transient effect with individuals getting re-infected after short periods. As was expected, HIV-1 negative individuals had normal CD4+ levels throughout the study. However, CD4+ levels among HIV-1 positives who started treatment were low at enrollment but increased significantly within the first month of treatment. From our association analysis, the decline in parasitemia among the HIV-1 positives on treatment was attributed to TS treatment and not increased CD4+ levels *per se*. Overall, this study highlights important interactions between HIV-1 and malaria that may inform future use of TS among HIV-infected patients in malaria endemic regions.

## Introduction

In areas endemic for both malaria and HIV-1, a number of studies have documented that malaria symptomatic individuals with HIV-1 co-infection have higher parasitemias than individuals without HIV-1 infection, and that HIV-1 infected individuals experience transient increases in viremia when suffering from an episode of clinical malaria (Patnaik et al., 2005; Hewitt et al., 2006; Laufer et al., 2006; Van Geertruyden et al., 2008). The first published clinical interaction between malaria and HIV-1 was from a 1987-89 trial in Malawi in which HIV-1 infection was associated with a diminished ability of pregnant women to control maternal *P. falciparum* infection, significantly increasing the risk of placental transmission of parasites to children from co-infected mothers (Steketee et al., 1996b; Steketee et al., 1996a). Since then, other interactions between the virus and clinical malaria episodes in different HIV-1 patient groups have been documented (reviewed in Hewitt et al., 2006). For example, increased parasitemia and more frequent clinical episodes of malarial fever have been associated with decreased CD4+ T cell counts in HIV-1 symptomatic individuals in Uganda and Malawi, a trend that was not observed in individuals earlier in the courses of their HIV-1 infection (Whitworth et al., 2000; French et al., 2001; Patnaik et al., 2005). In addition, in a study in Uganda in which HIV-1 infected and uninfected participants were scheduled to attend routine clinic visits every 3 months and whenever they fell ill, HIV-infected adults presented with nearly twice as many episodes of parasitemia and more clinical malaria episodes relative to HIV-negative adults, indicating that both asymptomatic and symptomatic malaria were increased by co-infection (Whitworth et al., 2000). Results from a community cohort in Kenya demonstrated similar findings: adults with advanced HIV-1 (lower CD4+ T-cell counts) and malaria had higher parasitemias and were at increased risk for clinical malaria (Marsh and Kinyanjui, 2006). These and other previous studies on HIV-malaria co-infection have focused primarily on symptomatic malaria cases (reviewed in Flateau et al., 2011).

To expand this understanding of HIV-malaria co-infection, we have specifically evaluated the epidemiological impact of HIV-1 infection on asymptomatic *sub-clinical* parasitemia, infections that could represent a significant source of transmission. In areas where malaria is endemic, many people can harbor parasite infection without overt illness (i.e., asymptomatic infection). Notably, our earlier cross-sectional studies showed that apparently healthy HIV-1 positive adults exhibited higher asexual malarial parasitemias (Kifude et al., 2021) and gametocytemias (Stiffler et al., 2021) compared to adults with malaria only. In the present study, we have extended these observations by recording the incidence, persistence, and prevalence of asymptomatic parasitemia over time in HIV-1 co-infected volunteers and in volunteers without HIV-1 infection.

Currently, the standard of care in Kenya for HIV-infected individuals includes a Nucleoside Reverse Transcriptase Inhibitor (NRTI) and two Non-Nucleoside Reverse Transcriptase Inhibitors (NNRTIs) as a first line treatment (Ministry of Health of Kenya, 2018). In all regimens, a fixed dose of multivitamin supplementation and trimethoprim-sulfamethoxazole (TS) are prescribed (Ministry of Health of Kenya, 2018). TS is used as a prophylaxis for opportunistic infections including malaria and is effective in reducing morbidity and mortality in people infected with HIV (Anglaret et al., 1999; Wiktor et al., 1999; Mermin et al., 2004). Second line ART protease inhibitors (PIs), not yet in widespread use in Kenya and most malarious countries, have also been shown to inhibit parasite growth *in vitro* (Parikh et al., 2005; Andrews et al., 2006). Data from a randomized controlled trial (RCT) designed to evaluate the impact of PI-based ART on malaria in Ugandan children demonstrated use of PI-based ART reduced the incidence of malaria by 41% compared to non-PI-based ART, largely attributable to a significant reduction in the risk of recurrent malaria following Artemether/Lumefantrine (AL) treatment (Achan et al., 2012).

Despite the impact of HIV therapeutics on malaria described above, some studies have shown somewhat conflicting impacts on malaria in endemic regions. In one study, Nigerian children on ART had a higher prevalence of asymptomatic parasitemia and higher density parasitemias than did children who were not on ART (Adetifa et al., 2008). Two other reports from retrospective analyses of a randomized controlled trial in women in sub-Saharan Africa failed to demonstrate a clinically relevant benefit in reduced malaria episodes in protease inhibitor (PI)-based ART compared to non-PI-based ART (Porter et al., 2012; Skinner-Adams et al., 2012).

Thus, there is still need for in-depth understanding of the impact of ART on malaria and more specifically on transmission in endemic areas with high use of both HIV treatment and antimalarials. Further, still fewer studies have examined the impact of ART and TS on asymptomatic parasitemia, which accounts for the majority of malaria infections in endemic regions. To address this gap in knowledge, we completed a longitudinal study of newly diagnosed HIV-1 positive patients before and after initiation of treatment along with HIV-negative individuals and HIV-1 positive individuals stable on ART to determine the extent to which initiation of ART and TS treatment and associated immune reconstitution altered the asymptomatic carriage of malaria parasites.

## Materials and methods

### Study site and participants

This longitudinal observational study was conducted in Kombewa, Kisumu West, located in the Lake Victoria Basin of Western Kenya. Malaria parasite transmission in this region occurs throughout the year with an estimated entomological inoculation rate for *P. falciparum* of 31.1 infectious bites per person per year (Ndenga et al., 2006). Our previous cross-sectional surveillance study of adults from 2015 and 2018 revealed no significant differences in prevalence of parasitemia during the study period (Kifude et al., 2021). In this study, enrollment of volunteers occurred between August 2018 and November 2019. Six monthly follow-up visits were scheduled through May 2020. Details of the study including a flowchart of sampling events, inclusion and exclusion criteria have been described in a previous publication by our group (Oyieko et al., 2022). Briefly, a total of 300 volunteers (>18 years old) were recruited into the study. Of these, 102 were HIV-1 negative, 106 were newly diagnosed with HIV-1 and immediately started on ART and TS, and 92 volunteers were HIV-1 positive but already stable on ART. The first line treatment for newly diagnosed HIV-1 participants was lamivudine, tenofovir, disoproxil fumerate, and dolutegravir (3TC+TDF+DTG), in addition to multivitamin supplementation and TS. Participants self-presented for voluntary HIV-1 testing and counseling at the HIV-1 Testing and Counseling (HTC) Center, Kisumu West Hospital, Kombewa sub-county, Nyanza Province, Kenya, or at an HTC Center in the Kisumu West District associated with the Kenya Medical Research Institute (KEMRI)/Walter Reed Project (WRP) PEPFAR program. HIV testing was performed according to the approved national testing algorithm and per Kenyan government HIV testing standard operating procedures (SOPs); both HIV-1 positive and HIV-1 negative participants were recruited from those presenting for voluntary testing. The 92 volunteers already on ART and TS were recruited from the PEPFAR program in Kisumu West District. All volunteers provided informed consent based on study details and blood sampling required for the longitudinal study. No specific malaria screening was performed on the volunteers. However, following consent, a malaria rapid diagnostic test (RDT) was administered using the Para screen Pan/*Pf* RDT (Zephyr Biomedicals, Verna, Goa, India) and appropriate treatment was provided, if needed, following Kenya Ministry of Health (MoH) malaria treatment guidelines. Any malaria RDT positive participants were treated with a three day course of AL in accordance with Kenyan MoH guidelines (Ministry of Health of Kenya, 2016).

### Sample collection

A baseline blood sample (50 mL) was collected from all 300 volunteers at enrollment (Month 0, M0). This was followed by a monthly blood collection of 20mL for six consecutive months (M1 to M6). For the HIV-1 positive newly diagnosed group, an additional 20 mL blood was collected at weeks 1 and 2 (Wk 1 and Wk 2) after initiation of ART and TS treatment to describe shorter term malaria kinetics immediately after initiation of HIV therapy. For all participants, complete blood count (CBC; Coulter AcT5Diff AL hematology analyzer, Beckman Coulter Diagnostics), CD4+ T cell count (PIMA CD4 analyzer, Abbott), and malaria RDT tests were performed at M0 and during all scheduled monthly follow-up visits. In addition, 50 μl blood samples were collected on Whatman^®^ 903 Protein Saver filter paper cards (GE Healthcare Life Sciences, Chicago, IL, USA), immediately air-dried, and stored at -80°C for subsequent qPCR assays for malaria parasitemia and speciation as described (Kifude et al., 2021).

### Data analysis

Data analyses were performed using GraphPad Prism version 8.4.1 (GraphPad, San Diego, CA, USA) and Stata version 16 (Stata Corp, College Station, TX, USA). Variables were summarized as frequencies, percentages, ranges and mean ranges as appropriate. All continuous variables such as parasitemia (*18S* copy numbers/μl), CD4+ T cells, age, and CBC values were tested for normality using the D’Agostino & Person test of normality. Thereafter appropriate tests such as one-way ANOVA and Kruskal Wallis were selected to compare means across groups with posthoc tests for multiple comparisons between the groups. Kaplan-Meier analysis was used to describe time to asymptomatic parasitemia and comparisons were made using the log-rank test. Correlation analysis between different variables was done using Pearson’s correlation coefficient. The level of significance for all analyses was set at α = 0.05.

### Ethical considerations

Ethical approvals for human use were granted by the Ethical Review Committee of the Kenya Medical Research Institute, Nairobi, Kenya (SSC# 3606), and the Walter Reed Army Institute of Research Institutional Review Board, Silver Spring, Maryland, USA (WRAIR # 2346).

## Results

### Patient demographics

A total of 300 volunteers including HIV-1 negative (n=102), HIV-1 positive newly diagnosed (n=106), and HIV-1 positive stable on ART and TS (n=92) were enrolled and followed for six months. Of the 300 participants, 54% were females (162/300) and 46% were males (138/300). There were no differences in gender distribution across the three study groups (*p*>0.05) (**Table 1**). The average age of the volunteers was 33 with a range of 18 to 60 years. Participants age varied significantly across the three groups (*p*<0.0001, one-way ANOVA with multiple comparison by Tukey’s test), with the HIV-1 negative group being the youngest (median=28), followed by the HIV-1 positive newly (Median=33) diagnosed while the HIV-1 positive on treatment were the oldest (median=39) (**Table 1**). At enrollment, all 300 volunteers were tested for malaria by RDT and all positive cases treated with a three day regimen of AL according to Kenya MoH guidelines ( (Ministry of Health of Kenya, 2016). RDT positivity rates for all the groups was determined (**Table 1**). At enrollment, the RDT positivity test was significantly higher (*p*=0.0001) in both the HIV negative group (22.5%) and HIV positive before initiation of treatment (25.4%) compared to the participants who were already on ART (2.2%). Analysis was done on Complete Blood Count (CBC) parameters at enrollment. Hematological parameters varied across the three groups with significant differences being observed for WBCs, monocytes, hemoglobin, platelets and RBCs (**Table 1**). However, there were no differences in granulocytes across the three groups.

**Table 1:** Baseline characteristics of volunteers at enrollment.

Parameter	Ratios (percentages), Means (SD) or Median (IQR)	P-value for Multiple comparison tests	P value for posthoc multiple comparisons
Gender (Female)			
1) HIV-1 Negative	56/102 (54.9%)	0.4039^a^	N/A
2) HIV-1 Positive Newly Diagnosed	54/106 (50.9%)		
3) HIV-1 Positive On Treatment	52/92 (56.5%)		
Age			
1) HIV-1 Negative	28 (IQR 23-35)	0.0001^a^	1 vs 2 *p*=0.0003
2) HIV-1 Positive Newly Diagnosed	33(IQR 27-39)		2 vs 3 *p*=0.0064
3) HIV-1 Positive On Treatment	39(IQR 33-44)		1 vs 3 *p*<0.0001
Malaria Parasitemia Positivity by RDT			
1) HIV-1 Negative	23/102 (22.5%)	0.0001^a^	1 vs 2 NS
2) HIV-1 Positive Newly Diagnosed	27/106 (25.4%)		2 vs 3 *p*=0.0003
3) HIV-1 Positive On Treatment	2/92 (2.4%)		1 vs 3 *p*=0.0008
White Blood cells (x 10^9/L)			
1) HIV-1 Negative	5.568 (SD 1.57)	0.0017^a^	1 vs 2 *p*=0.0013
2) HIV-1 Positive Newly Diagnosed	4.989 (SD 2.06)		2 vs 3 NS
3) HIV-1 Positive On Treatment	5.052 (SD 1.34)		1 vs 3 NS
Neutrophils (%)			
1) HIV-1 Negative	45.68 (SD 10.51)	0.4131^b^	N/A
2) HIV-1 Positive Newly Diagnosed	46.52 (SD 12.53)		
3) HIV-1 Positive Newly Diagnosed	44.49 (SD 11.23)		
Monocytes (%)			
1) HIV-1 Negative	3.46 (SD 1.74)	0.0003^a^	1 vs 2 *p*=0.0033
2) HIV-1 Positive Newly Diagnosed	4.72 (SD 3.12)		2 vs 3 *p*=0.0008
3) HIV-1 Positive On Treatment	3.36 (SD 1.66)		1 vs 3 NS
Hemoglobin (g/L)			
1) HIV-1 Negative	13.75 (SD 1.90)	0.0018^b^	1 vs 2 *p*=0.0013
2) HIV-1 Positive Newly Diagnosed	12.52 (SD 2.36)		2 vs 3 NS
3) HIV-1 Positive On Treatment	13.13 (SD 2.34)		1 vs 3 NS
Platelets (fL)			
1) HIV-1 Negative	228.6 (SD 63.34)	0.0104^a^	1 vs 2 NS
2) HIV-1 Positive Newly Diagnosed	244.1 (SD 98.16)		2 vs 3 NS
3) HIV-1 Positive On Treatment	257.9 (SD 76.35)		1 vs 3 *p*=0.0077
RBCs (x 10^12/L)			
1) HIV-1 Negative	5.04 (SD 0.59)	0.0003^a^	1 vs 2 *p*=0.0014
2) HIV-1 Positive Newly Diagnosed	4.78 (SD 0.69)		2 vs 3 NS
3) HIV-1 Positive Newly Diagnosed	4.79 (SD 0.65)		1 vs 3 *p*=0.008

D’Agostino & Pearson test of normality test showed that the data were not normally distributed. Pairwise comparison of means was performed with Kruskal Wallis^a^ test and posthoc Dunn’s multiple comparison test or with one-way ANOVA^b^ and posthoc Tukey’s correction.

Gender, age, malaria status by RDT, and hematological parameters were compared between (i) HIV-1 negative, (ii) HIV-1 positive newly diagnosed, and (iii) HIV-1 positive on treatment. Data for baseline parameters (Month 0) were analyzed by appropriate ANOVA test. Any parameter with statistical difference across the three groups (in bold) was subjected to a posthoc analysis for multiple comparisons using Tukey’s test (one-way ANOVA) or Dunn’s Test (Kruskal Wallis).

### Temporal prevalence of asymptomatic malarial parasitemia by 18S qPCR and RDT

**Figure 1:**
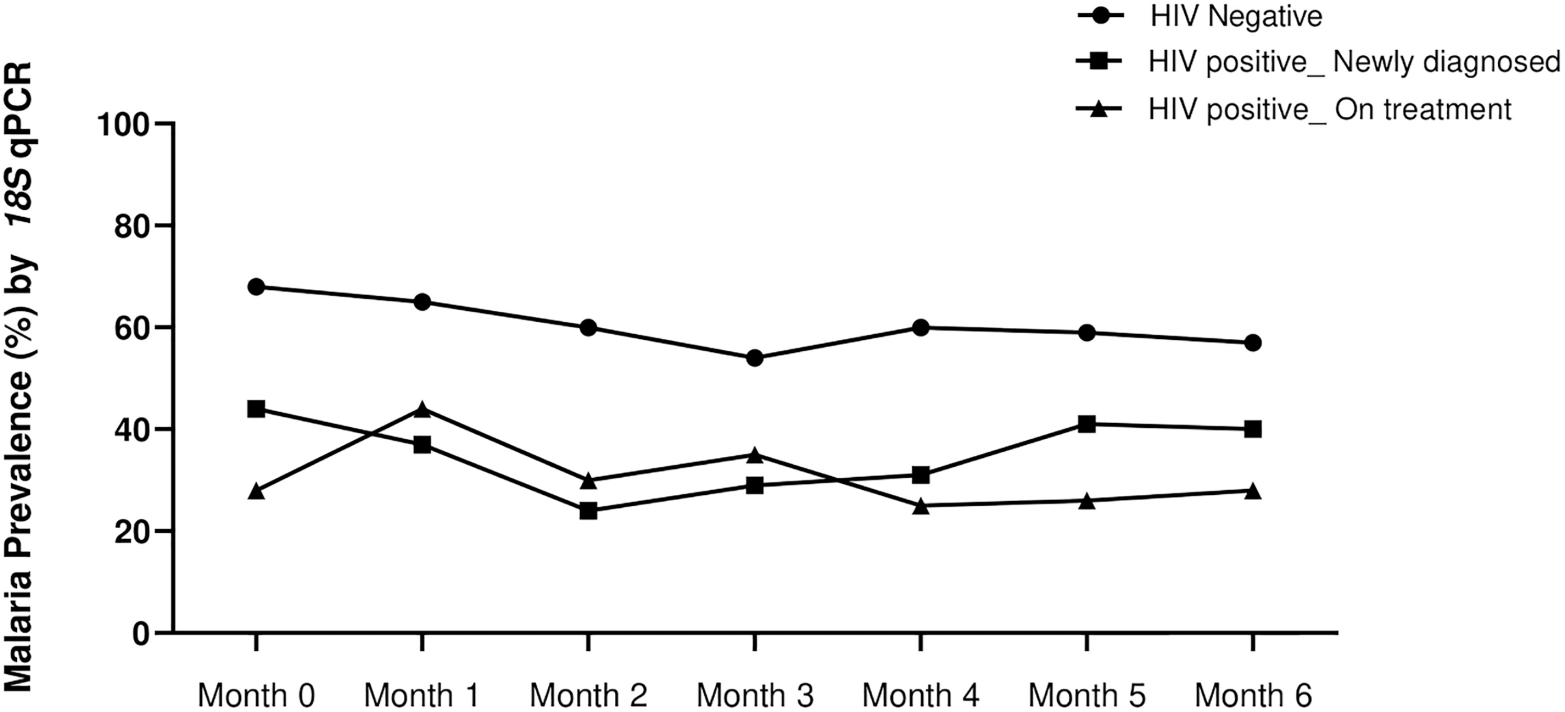
Temporal malaria positivity rate as determined by *18S* genus qPCR assay at enrollment (month 0) and during 6 months follow up (months 1-6). Prevalence of asymptomatic parasitemia was highest in the HIV-1 negative volunteers (black circles) at all time-points (*p*=0.0001, one way ANOVA with Tukey’s posthoc analysis). The prevalence in HIV-1 positive newly diagnosed (squares) and HIV-1 positive on treatment (triangles) was not different (*p*=0.5556).

A total of 1836 blood samples were collected from all 300 volunteers during all visits. The overall study retention rate was 79.4% while the average number of visits per participant was six. Of these samples, 1835 were successfully tested for *Plasmodium* spp. by our genus specific *18S* qPCR assay as previously described (Kifude et al., 2021). Malaria prevalence based on *18S* qPCR assay was significantly higher in the HIV-1 negative group (61.39%, 345/562, *p*=0.0001, one way ANOVA with Tukey’s correction) compared to both the HIV-1 positive newly diagnosed 36.52 (278/761) and HIV-1 positive on treatment 31.45% (161/512). There was no difference between the two latter groups (*p*=0.5556). This trend was observed at enrollment and during most of the subsequent follow-up visits (**Figure 1**). As expected, the highest proportion of infections over all visits were identified as *P. falciparum* (33.2%, 260/784), while *P. malariae* and *P. ovale* accounted for 5.6% (44/784) and 1.6% (13/784) of infections, respectively (**Supplementary** **Figure 1**). The distributions of infecting parasite species varied slightly across the three groups, albeit not significantly. *P. falciparum* and *P. malariae* were evident in HIV-1 negative volunteers as single and mixed infections. Conversely, *P. ovale* was detected as a single infection in HIV-1 positive volunteers: 11 of 13 P*. ovale* positive samples (84.6%) were detected in the absence of other infecting parasite species. As in our previous cross sectional study (Kifude et al., 2021), and like other previous studies within the region (Howes et al., 2011), no *P. vivax* infections were detected in this population.

**Figure 2:**
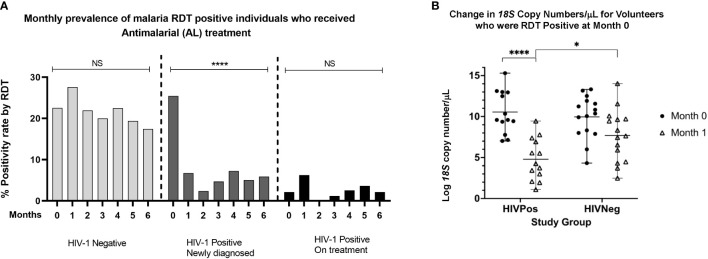
**(A)** Monthly prevalence rates for individuals who were RDT positive and who received AL treatment across the three study groups. Significant reductions in parasite prevalence were seen among the HIV-1 positive newly diagnosed following initiation of ART/TS treatment but not among the HIV-1 negative group and the HIV-1 positives on treatment (one way ANOVA with Tukey’s HSD posthoc analysis. **(B)** Comparison of *18S* copy numbers for HIV positive and HIV negative volunteers who were RDT positive at month 0 and month 1. There were significant differences in *18S* copy numbers between individuals who received both antimalarial and HIV treatment versus those who received antimalarial treatment only. ****p < 0.0005, *p < 0.05. NS means not significant.

At enrollment, 52 of the 300 volunteers (17.3%) were malaria RDT positive and hence received AL treatment. It was expected that antimalarial treatment at any given visit would have an impact on parasite densities at subsequent visits. When stratified by groups, RDT positivity rates were similar for the HIV-1 negative group and HIV-1 positive newly diagnosed group at enrollment; 22.5% (23/102) versus 25.4% (27/106) respectively (**Figure 2A**). HIV-1 positive participants who were stable on ART had the lowest malaria positivity rate of 2.2% (2/92) at the time of enrollment. In the subsequent monthly follow-up visits, malaria RDT positivity rate among the HIV-1 negatives remained high (>17%) throughout the 6 months of follow up, suggesting that AL treatment at each visit did not affect malaria prevalence in the subsequent visits. However, in the newly diagnosed HIV positive group, malaria RDT positivity rate dropped significantly within the first month from 25.5% to 6% and remained relatively low (> 6%) throughout the entire follow-up period (**Figure 2A**), suggesting that the drop was driven by initiation of HIV treatment and not specific antimalarial treatment. Low positivity rates (<6%) were observed for the HIV-1 positives who were stable on ART throughout the entire study period. We further examined the impact of antimalarial treatment on the parasite load (*18S* copies/μl) in the context of initiation of ART/TS. Overall, RDT positive volunteers had significantly higher copy numbers (*p*<-.0001) than the RDT negatives (**Supplementary** **Figure 2**), affirming known differences in sensitivity of these methods (Kifude et al., 2021). In comparing HIV-1 negatives and HIV-1 positives that were RDT positive at enrollment, we observed that initiation of treatment was associated with reduced malarial *18S* copy numbers/μl between month 0 and month 1(*p*<0.0001) in HIV positive newly diagnosed volunteers on ART, TS and antimalarial relative to HIV negative volunteers on antimalarial treatment only (**Figure 2B**). Taken together, these findings show ART/TS to be effective in not only reducing monthly prevalence rates, but also the burden of asymptomatic parasitemia.

**Figure 3:**
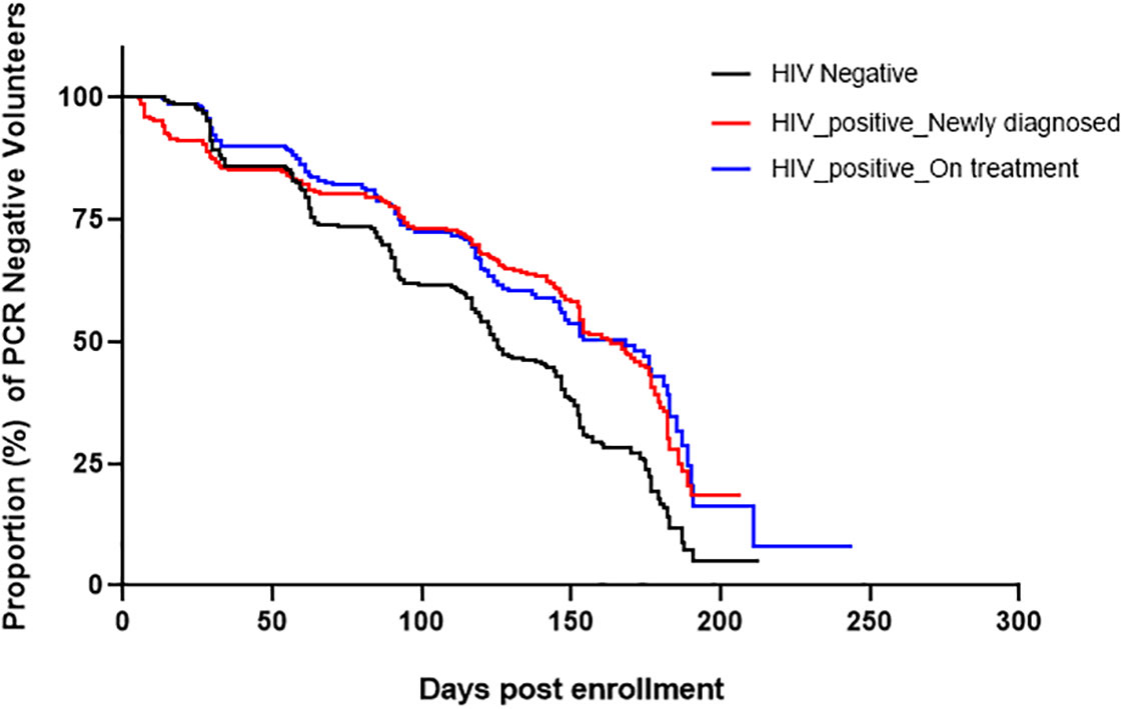
Kaplan-Meier curves showing time to infection post-enrollment. Only volunteers who were negative by *18S* PCR at enrollment were included in this analysis. The median time to infection for HIV-1 negative volunteers was significantly shorter 149 days, (Log-rank Mantel-Cox test; χ^2^(2) = 7.3, *p*=0.00136) while that of HIV-1 positive newly diagnosed and HIV-1 positive on treatment were 171 and 177 days, respectively.

Next, we examined the association of HIV treatment with incidence of asymptomatic parasitemia. Participants who were *18S* PCR negative at enrollment exhibited significant differences by study group in time to detection of asymptomatic parasitemia post-enrollment by Kaplan-Meier analysis. As shown in **Figure 3**, the median time to the first asymptomatic infection for the HIV-1 negative group was significantly shorter 149 days (*p*=0.00136) compared to HIV-1 newly diagnosed (171 days) and HIV-1 on ART (177 days). There was no difference in the median time to infection between the latter two groups.

### Association of HIV diagnosis and treatment with 18S copy numbers over time

**Figure 4:**
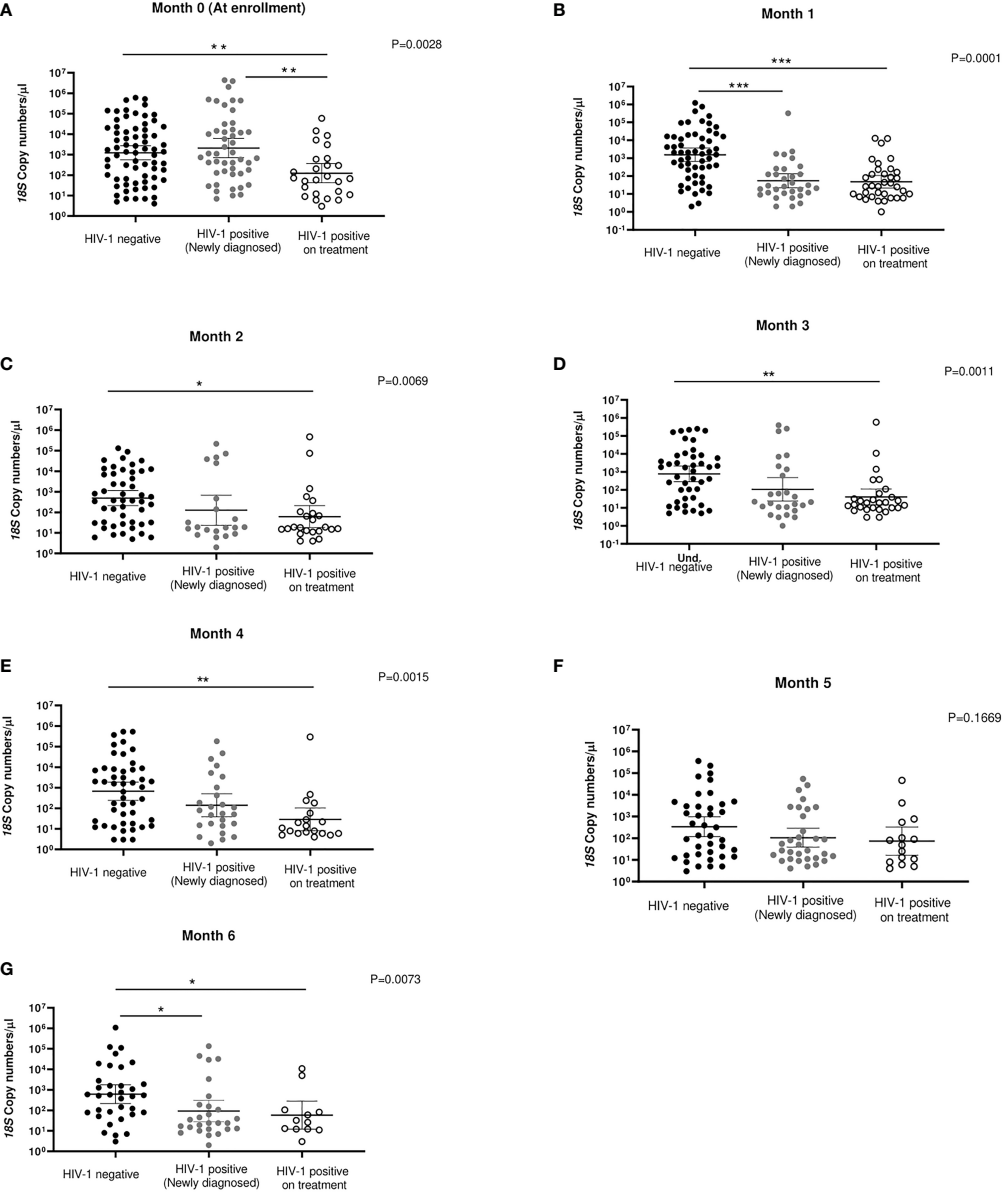
*18S* copy numbers/μl at enrollment (month 0) (Panel **A**) and at follow-up from months 1-6 (Panels **B-G**) in the three study groups. *p-*values at each time-point were determined using Kruskal Wallis test for multiple comparisons followed by Dunn’s Test for multiple comparisons. ***p<0.0005, **p<0.005, *p<0.05.

In order to determine the impact of ART/TS treatment on asymptomatic parasitemia, analysis of mean *18S* copy numbers/μl for the three groups at different monthly time-points was completed using non-parametric Kruskal Wallis test with Dunn’s posthoc test for multiple comparison. All time points, apart from month 5, showed significant differences in geometric mean *18S* copy numbers across the study groups, with significant differences in copy numbers between the HIV-1 negative group and the HIV-1 positive group who were stable on ART. At enrollment (Month 0), the mean *18S* copy numbers did not differ significantly between HIV-1 negative volunteers (geometric mean = 1234, CI 559-2728 copies/μL) and HIV-1 positive newly diagnosed volunteers (geometric mean 2096 CI 709-6191 copies/μL) before initiation of ART and TS (*p*=0.9998) (**Figure 4A**). However, at the second visit, one month after initiation of ART and TS treatment, *18S* copy numbers in HIV-1 positive newly diagnosed volunteers dropped significantly (*p*=0.0001; geometric mean 55, CI 22-136), approaching the level in HIV-1 positive volunteers stable on treatment (geometric mean = 48, CI 21-107). In contrast, copy numbers for HIV-1 negative volunteers remained significantly higher (**Figure 4B**) compared to the two HIV-positive groups (geometric mean = 1546, CI 657-3642) *p*<0.0005). For most remaining follow-up visits (months 2-6, except for month 5), there were significant differences across the three groups, with copy numbers being higher in the HIV-1 negative group compared to the two HIV-1 positive groups (**Figures 4C–G**).

**Figure 5:**
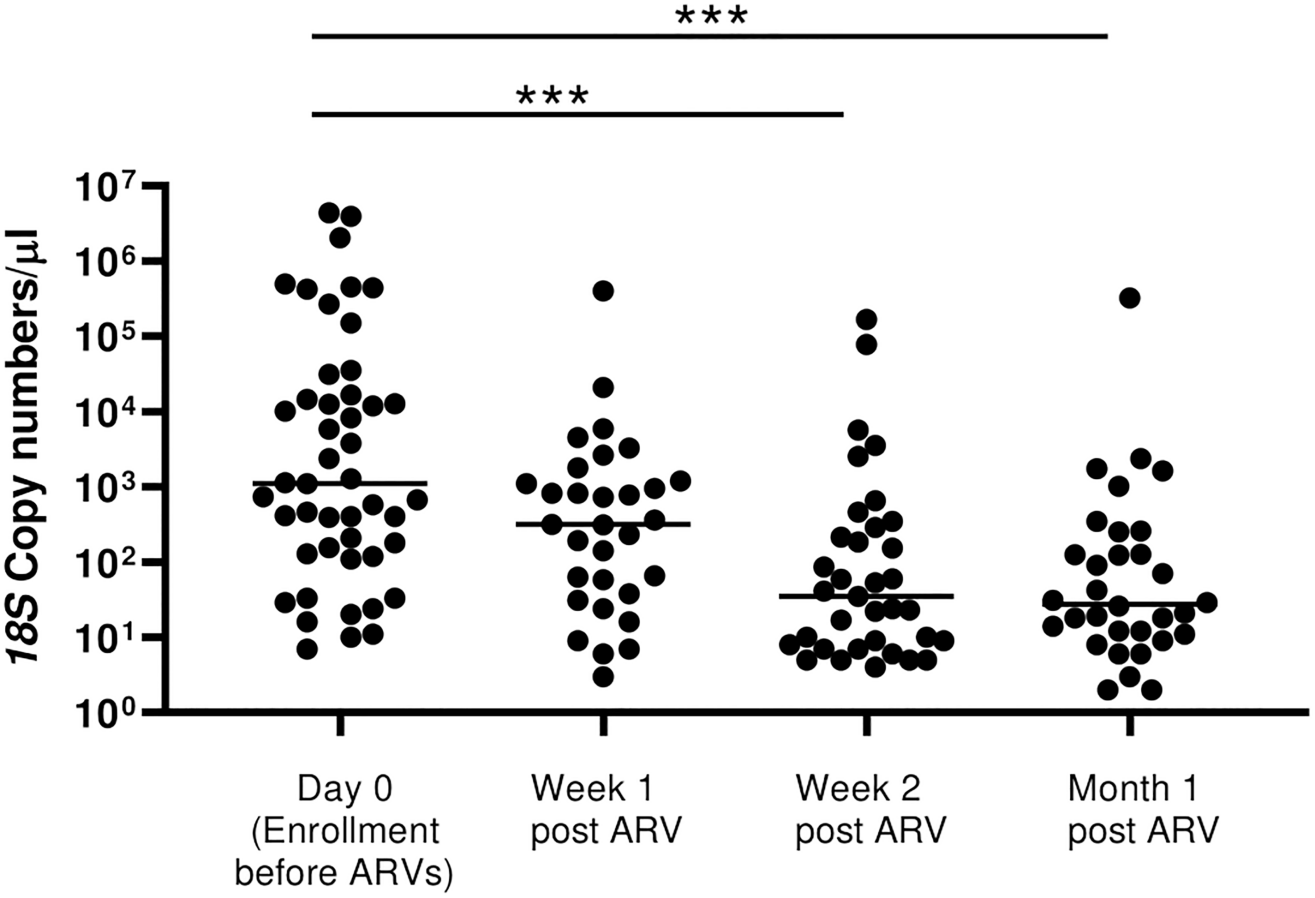
Rate of drop in *18S* copy numbers among all the HIV-positive newly diagnosed following initiation of ART. There was a stepwise drop in parasitemia at Week 1, Week 2 and at month 1 following initiation of ART/TS (*p*=0.0001 by Kruskal Wallis test). However, post-hoc analysis by Dunn’s test to compare the means showed significant differences between Day 0 with Week 2 (***p=0.0005) and between Day 0 with Month 1 (***p=0.0005).

Having observed a drop in *18S* copy numbers/μl within the first month of initiation of ART/TS among the newly diagnosed HIV-1 positives, we sought to describe the rate of drop in copy numbers using the samples collected at Week 1 and Week 2 following initiation of treatment that were available in this group only. There was a drop in 18S copy number by 85.8% from a geometric mean of 2096 to 297 *18S* copies/μL, after only one week of treatment (**Figure 5**). During the second week and after one month of follow-up, copy numbers had further dropped significantly by more than 96.7% relative to month 0 to a mean of 69.3 copies/μL and 55.15 copies/μL, respectively (*p*=0.0001).

### CD4+ T Cell densities associated with HIV-1 infection status and treatment

**Figure 6:**
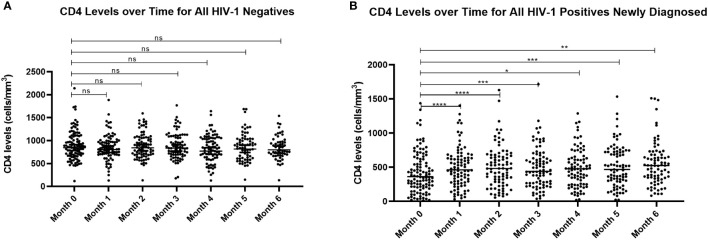
Comparison of CD4+ T cell counts for **(A)** HIV-1 negative volunteers and **(B)** HIV-1 positive newly diagnosed volunteers over time. Analysis by mixed model ANOVA with Dunnett’s multiple comparison test showed significant differences in CD4+ T cell counts for HIV-1 positive volunteers over time relative to month 0 (****p<0.0001, ***p<0.0005, **p<0.005, *p<0.05). There were no differences for the HIV-1 negative group. ns means not significant.

Current treatment guidelines recommend immediate initiation of ART/TS for all newly diagnosed HIV patients regardless of their CD4+ T cell counts (Ministry of Health of Kenya, 2018). For research purposes, we sought to determine the impact of treatment on CD4+ T cell levels and whether there were any associations with parasitemia. As expected, CD4+ T cell counts at enrollment were significantly higher (*p*=0.0001) in the HIV-1 negative group compared to HIV-1 newly diagnosed and those already on treatment. There were no differences in CD4+ T cell levels over time for HIV-1 negative volunteers (**Figure 6A**). However, as anticipated, in the newly diagnosed HIV-1 positives, CD4+ T cell counts were significantly increased after Month 0 (before treatment) and all the subsequent follow-up visits (*p*<0.05) (**Figure 6B**).

**Figure 7:**
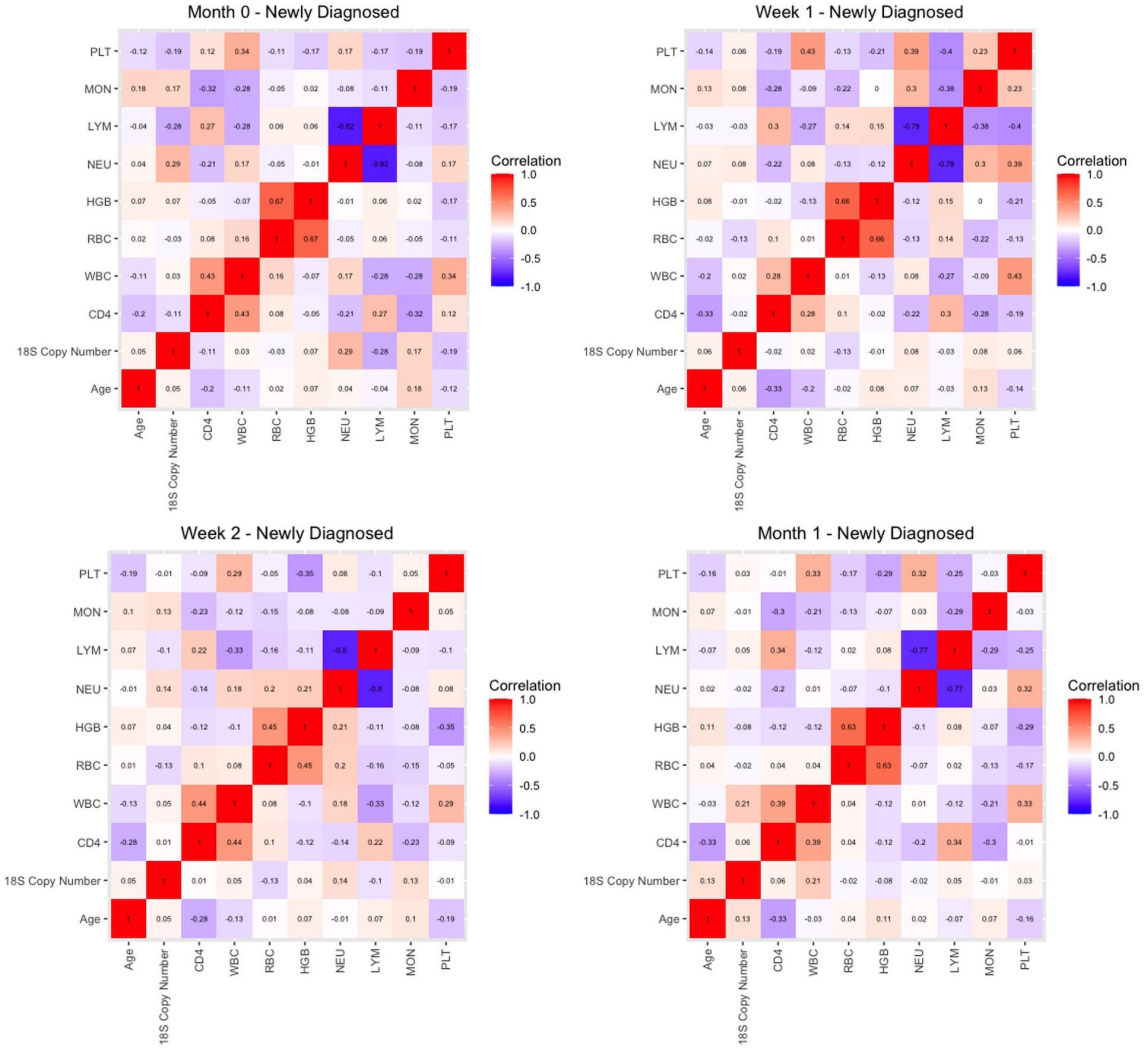
Correlation matrices for key parameters in HIV-1 newly diagnosed volunteers prior to initiation of ART and TS (month 0) and at week 1, week 2, and one month later.

Finally, we sought to determine whether malaria *18S* copy numbers for HIV-1 positive newly diagnosed volunteers were correlated with age and hematological parameters including CD4+ T cell counts through the first month of the study. Our goal was to establish whether any of these parameters could explain the decline in copy number following initiation of treatment. However, correlation matrices before treatment (M0) and after week 1 (Wk 1), Week 2 (Wk 2) and Month 1 (M1) showed no significant correlations among these parameters and *18S* copy numbers/μl (**Figure 7**).

## Discussion

In sub-Saharan Africa, where there is a geographical overlap of HIV-1 and malaria, available data suggest that the impacts of clinical co-infection are both synergistic and bidirectional (French et al., 2001; Laufer et al., 2006; Torrevillas et al., 2020; Kifude et al., 2021; Stiffler et al., 2021). In this context, numerous studies on the interactions of clinical malaria and ART in HIV-1 positive patients have been published (Fehintola et al., 2011; Flateau et al., 2011; Kasirye et al., 2016; Kasirye et al., 2017). Further, TS has been shown to ameliorate clinically apparent symptomatic malaria in HIV-infected patients (Omar et al., 2001; Sowunmi et al., 2005), but the impacts of treatment and/or immune reconstitution on asymptomatic malarial parasitemia have not been studied in detail. Accordingly, we sought to examine the associations between HIV-1 and treatment with ART/TS with asymptomatic parasitemia over a period of six months. To this end, we enrolled participants before initiation of ART and TS so that the impact of treatment on asymptomatic parasitemia could be ascertained. Our interest in the asymptomatic population stems from the fact that semi-immune individuals likely serve as the primary reservoir of malaria (Bousema et al., 2014). Thus, HIV-infected individuals from this population are predicted to have increased prevalence, frequency, duration, and/or intensity of asymptomatic parasitemia. We observed declines in both prevalence and parasite density only among the HIV-positive volunteers on ART/TS treatment that appeared to be associated with HIV treatment rather than specific antimalarial treatment. (**Figures 2A, B**). Our findings suggest that the impact of AL treatment is quite transient and that individuals living in malaria endemic areas are quickly re-infected after treatment with antimalarials. Our data also support previous observations of a synergistic effect of antimalarials and ART/TS on parasitemia among the HIV-infected individuals (Kapito-Tembo et al., 2011).

These data, to our knowledge, are among the first to show this association under the current WHO HIV-1 treatment guidelines of “test and treat” (Ministry of Health of Kenya, 2018), particularly in an area where antifolate resistance is extremely common (Iriemenam et al., 2012; Juma et al., 2014). We can posit two hypotheses for this decline in parasitemia among the HIV-1 positive newly diagnosed volunteers who were initiated on ART and TS compared to the HIV-1 negative participants in an area where antifolate drug resistance in parasites is common (Iriemenam et al., 2012; Juma et al., 2014) **(** **Figures 4** and **5** **).** The first would implicate immune reconstitution (Bishop et al., 2016), while the second is based on the persistent metabolic cost of folate antagonists to genetically resistant parasites (Wang et al., 2004). To examine this first hypothesis, we evaluated the correlation between parasitemia and CD4+ T cell counts to determine whether this decline in parasitemia was associated with TS or immune reconstitution following treatment. While there was an increase in CD4+ T cell levels after initiation of treatment among newly diagnosed HIV positives, CD4+ T cell levels and parasitemia (*18S* copy numbers/μL) were not significantly correlated, somewhat favoring a role for TS versus immune reconstitution. Two previous studies (Polyak et al., 2016; Ottichilo et al., 2017) showed effects of TS on malaria parasitemia, with increased incidence of malaria following discontinuation of TS. However, both studies examined the impact of discontinued TS in patients receiving ART for 18+ months rather than effects of TS initiation on parasitemia. In our study, participants were enrolled at the time of diagnosis of HIV-1 infection so that the true incidence, malaria status and *18S* copy number over time could be ascertained. Enrollment at the time of diagnosis allowed for control of multiple parameters (HIV-1 status, CD4+ T cell counts, ART and TS treatment) that cannot be fully temporally defined in HIV-1 positive individuals already on ART and TS. Due to current treatment guidelines, we could not determine whether declines in *18S* copy number were due to TS or to ART (3TC+TDF+DTG). A recent rodent study provides some insight: analyses of the impact of current first-line antiretrovirals and the alternative combinations (18 compounds) on *P. berghei* revealed no effects on sexual and asexual parasite development (Azevedo et al., 2020), suggesting that the impact of first line HIV-1 treatment on malaria is driven by TS.

The clinical benefits of TS prophylaxis in controlling opportunistic infections including falciparum malaria in HIV-infected individuals are clear. Previous studies have shown that the use of TS decreases morbidity, mortality and hospitalizations among HIV-infected patients (Wiktor et al., 1999, Chintu et al., 2004, Lowrance et al., 2007). In addition, the relevance of TS is supported by studies where discontinuation of TS in ART-treated population resulted in increased prevalence of clinical malaria, diarrhea and pneumonia (Campbell et al., 2012). There are, however, concerns about the development of TS resistance associated with this usage. Thus, it may be important to monitor the impacts of TS on malaria for longer periods of time. In this context, analyses of antifolate resistance would be informative. A study in Western Kenya (Juma et al., 2018) showed that TS does not select for Sulfadoxine/Pyrimethamine (SP)-resistant *P*. *falciparum*, but instead, lowers the overall incidence of SP-resistant parasites. Such findings need further validation, particularly in settings where SP continues to be used in intermittent preventive treatment (IPT) and where the dihydrofolate reductase (dhfr)/dihydropteroate synthetase (dhps) quintuple mutant has been associated with SP treatment failure (Bwijo et al., 2003). The short-term and longer term evolution of the HIV-1 strains circulating at the time of diagnosis and over the course of six months would also provide insights regarding the rate at which antiretroviral drug resistance is emerging in this population.

Importantly, we note that while TS appears to ameliorate asymptomatic parasitemia in HIV-infected patients, its continued use could enhance the prevalence and intensity of asymptomatic gametocytemia (Sowunmi et al., 2005). This could have the unwanted effect of confounding malaria control efforts by enhancing parasite transmission. In conclusion, the data presented here demonstrate the impact of TS on asymptomatic malaria during the early phases of HIV-1 treatment. We are examining patterns of gametocytemia associated with TS (Roberds et al., 2022), with additional studies focused on patterns of parasite transmission.

## Data availability statement

The original contributions presented in the study are included in the article/**Supplementary Material**. Further inquiries can be directed to the corresponding author.

## Ethics statement

The studies involving human participants were reviewed and approved by Ethical Review Committee of the Kenya Medical Research Institute, Nairobi, Kenya (SSC# 3606). Walter Reed Army Institute of Research Institutional Review Board, Silver Spring, Maryland, USA (WRAIR # 2346).

## Authors contributions

CK carried out the qPCR assays, performed statistical analysis and drafted original manuscript. AR assisted in data analysis and review of manuscript. JO, JW and NC supervised study activities in Kombewa, Kenya. SOt and SOc contributed to sample collection and analysis in Kenya. JH and HS reviewed the manuscript. SL and VS conceived and designed the longitudinal study, reviewed and revised the manuscript. All authors contributed to the article and approved the submitted version.

## Funding

This work was supported by NIH NIAID R01 AI104423 (VAS, SL).

## Acknowledgement

We thank the Kombewa Clinical Research Center including counselors and clinical laboratory staff for their help in sample collection and storage. Special thanks to the staff of Kombewa HIV-1 Testing and Counseling Center and the Kisumu West District WRP/KEMRI PEPFAR Program for their considerable help with this study. We also thank the staff of KEMRI/USAMRD-A Basic science Laboratory-Kondele and the Entomology lab-Kisian for supporting this project.

## Conflict of interest

The authors declare that the research was conducted in the absence of any commercial or financial relationships that could be construed as a potential conflict of interest.

## Publisher’s note

All claims expressed in this article are solely those of the authors and do not necessarily represent those of their affiliated organizations, or those of the publisher, the editors and the reviewers. Any product that may be evaluated in this article, or claim that may be made by its manufacturer, is not guaranteed or endorsed by the publisher.

## Author disclaimer

The contents, views and opinions expressed in this publication are those of the authors and do not necessarily reflect the official policy or position of Uniformed Services University of the Health Sciences. Mention of trade names, commercial products and organizations does not imply endorsement by the U.S. Government. Material has been reviewed by the Walter Reed Army Institute of Research and the Uniformed Services University. There is no objection to its presentation and/or publication. The opinions or assertions contained herein are the private views of the authors and are not to be construed as official or as reflecting true views of the Department of the Army or the Department of Defense. The investigators have adhered to the policies for protection of human subjects as prescribed in AR 70–25.
