# Supplementary material for: Initiation of anti-retroviral/Trimethoprim-Sulfamethoxazole therapy in a longitudinal cohort of HIV-1 positive individuals in Western Kenya rapidly decreases asymptomatic malarial parasitemia
Source: Front Cell Infect Microbiol. 2022 Nov 24;12:1025944. doi: 10.3389/fcimb.2022.1025944 (PMC9729353; doi:10.3389/fcimb.2022.1025944)
Supplement: Supplementary file 1 [file DataSheet_1.docx]

**Supplementary Material**


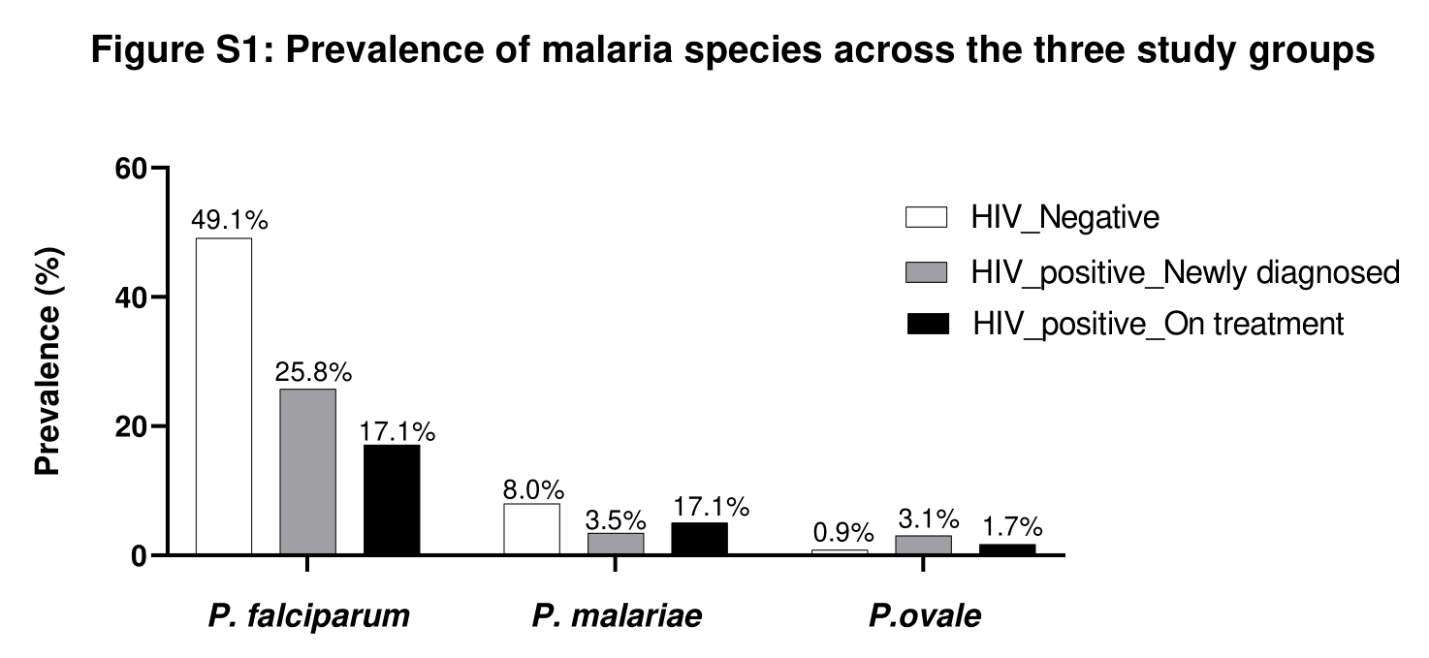


**Supplementary Figure 1:** Prevalence of malaria species at enrollment across the three study groups.


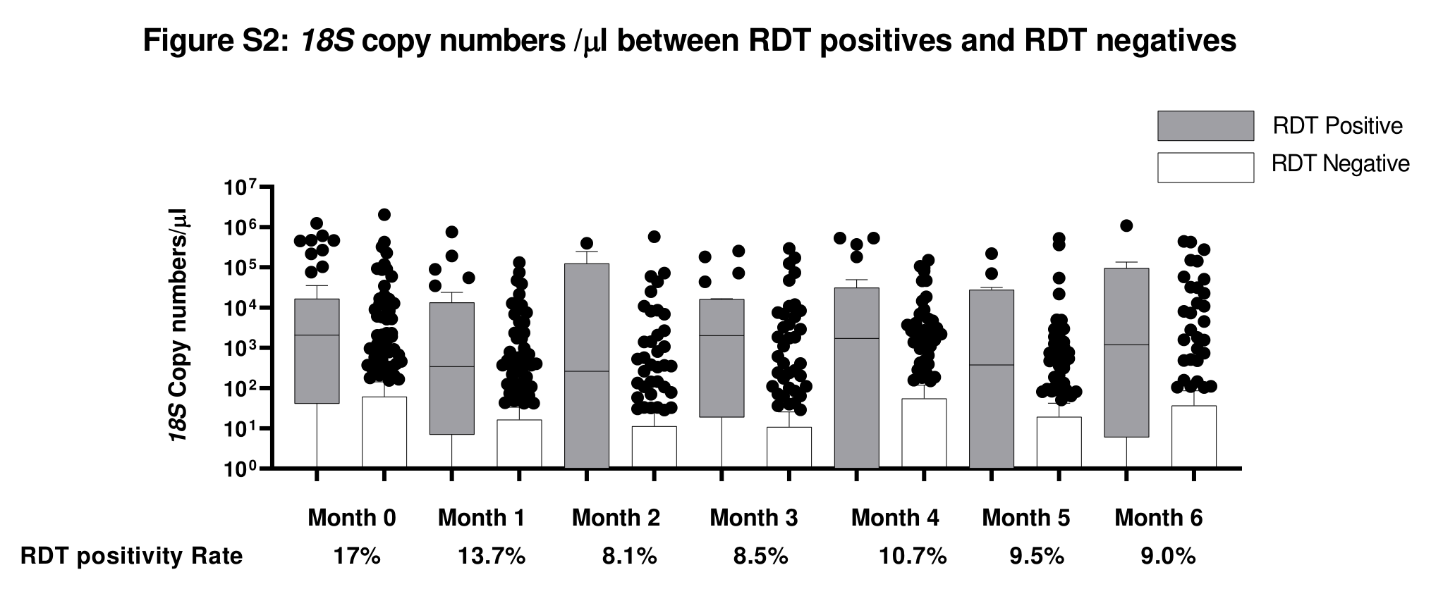


**Supplementary Figure 2:** Boxplots and Tukeys whiskers showing median 18S copy numbers per/ μl between volunteers that were RDT positive and those that were RDT negative. The monthly malaria positivity rate by RDT is shown below the graph.
